# Increases in depression and anxiety symptoms in adolescents and young adults during the COVID-19 pandemic

**DOI:** 10.1017/S0033291720005358

**Published:** 2021-01-13

**Authors:** Mariah T. Hawes, Aline K. Szenczy, Daniel N. Klein, Greg Hajcak, Brady D. Nelson

**Affiliations:** 1 Stony Brook University, Department of Psychology, Stony Brook, NY, USA; 2 Florida State University, Department of Psychology, Tallahassee, FL, USA

**Keywords:** anxiety, COVID-19, depression, pandemic, youth

## Abstract

**Background:**

The coronavirus [coronavirus disease 2019 (COVID-19)] pandemic has introduced extraordinary life changes and stress, particularly in adolescents and young adults. Initial reports suggest that depression and anxiety are elevated during COVID-19, but no prior study has explored changes at the *within*-person level. The current study explored changes in depression and anxiety symptoms from before the pandemic to soon after it first peaked in Spring 2020 in a sample of adolescents and young adults (*N* = 451) living in Long Island, New York, an early epicenter of COVID-19 in the U.S.

**Methods:**

Depression (Children's Depression Inventory) and anxiety symptoms (Screen for Child Anxiety Related Symptoms) were assessed between December 2014 and July 2019, and, along with COVID-19 experiences, symptoms were re-assessed between March 27th and May 15th, 2020.

**Results:**

Across participants and independent of age, there were increased generalized anxiety and social anxiety symptoms. In females, there were also increased depression and panic/somatic symptoms. Multivariable linear regression indicated that greater COVID-19 school concerns were uniquely associated with increased depression symptoms. Greater COVID-19 home confinement concerns were uniquely associated with increased generalized anxiety symptoms, and decreased social anxiety symptoms, respectively.

**Conclusions:**

Adolescents and young adults at an early epicenter of the COVID-19 pandemic in the U.S. experienced increased depression and anxiety symptoms, particularly amongst females. School and home confinement concerns related to the pandemic were independently associated with changes in symptoms. Overall, this report suggests that the COVID-19 pandemic is having multifarious adverse effects on the mental health of youth.

## Introduction

The novel coronavirus [coronavirus disease 2019 (COVID-19)] pandemic has disrupted the lives of individuals around the world. In the U.S., city and state-wide shelter-in-place orders were implemented across the country as early as March 2020, forcing most Americans to adjust to new circumstances (e.g. studying and working from home), take on new roles (e.g. caretaker, teacher), and deal with new or exacerbated hardship (e.g. job loss, reduced income, illness, social isolation, and restricted mobility). The nature and full extent of the impact of COVID-19 is only beginning to be understood as this unprecedented crisis unfolds.

An emerging literature suggests that depression and anxiety symptoms may be elevated during the COVID-19 pandemic, and that certain populations (e.g. women, individuals living in areas with a high density of COVID-19 cases) are more vulnerable to worsening mental health during the pandemic (Torales, O'Higgins, Castaldelli-Maia, & Ventriglio, 2020; Vindegaard & Benros, 2020). However, there are many limitations to the extant literature, including widespread reliance on cross-sectional designs and limited assessment of experiences related to the pandemic that may moderate psychiatric symptoms.

The vast majority of studies of mental health during COVID-19 have been conducted in Asia and Europe, where the disease first spread. The U.S. has since emerged as one of the most highly impacted countries, with the greatest number of total COVID-19 infections and deaths by April 2020 (McNeil Jr., 2020). Three studies using probability based nationally representative samples provide initial evidence that symptoms and rates of depression and anxiety have increased in the U.S. during the pandemic (Ettman et al., 2020; Holman, Thompson, Garfin, & Silver, 2020; Twenge & Joiner, 2020). However, all three studies compared rates between cohorts assessed before *v.* during COVID-19 or earlier *v.* later periods of the pandemic, so change could not be assessed at the *within*-person level. Additionally, the timing and severity of COVID-19 infections and local government responses have varied widely across the U.S., so substantial differences at the regional level are folded together in these nationally representative samples (Jacobson et al., 2020).

Young adults and adolescents may be especially vulnerable to mental health consequences of the COVID-19 pandemic (Gruber et al., 2020), In Spring 2020, nearly all education in the U.S. transitioned to remote learning and colleges across the country closed their dorms, forcing students to move, typically back home with their families, and limiting social interaction with peers. A recent review on the relationship between mental health and loneliness/social isolation in children and adolescents warned that COVID-19 social distancing measures may be particularly detrimental for youth (Loades et al., 2020). Further, student status and younger age have been associated with worse mental health during COVID-19 in Asian samples (Huang & Zhao, 2020; Wang et al., 2020).

A handful of longitudinal studies on adolescents and young adults have found that symptoms of anxiety and depression increased from before the COVID-19 pandemic (Elmer, Mepham, & Stadtfeld, 2020; Li, Cao, Leung, & Mak, 2020; Magson et al., 2020; Saraswathi et al., 2020). Of these, just one study was conducted in the U.S. Lee, Cadigan, and Rhew (2020) found that symptoms of depression and loneliness increased from right before (January 2020) to during (April/May 2020) the COVID-19 pandemic in a sample of young adults (aged 22–29) living in Seattle, Washington.

The current study explores the impact of the COVID-19 pandemic on depression and anxiety symptoms in adolescents and young adults living in Long Island, New York. New York state was one of the first regions in the U.S. to be severely impacted by the pandemic, reaching nearly 300 000 cases and over 18 000 deaths from COVID-19 by the end of April 2020, the highest rate per capita of any state at the time (Centers for Disease Control, 2020). Moreover, most cases were concentrated in the New York metropolitan area, including Long Island.

**Fig. 1.**
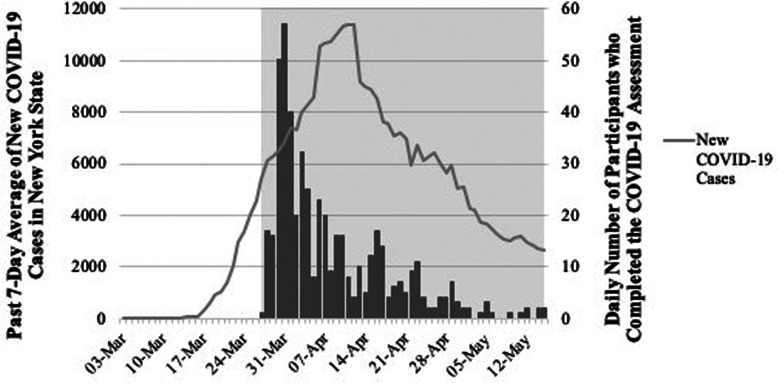
Past 7-day averages of new COVID-19 cases in New York State between March 3rd, 2020 and May 31st, 2020 (left y-axis, black line), and number of participants who completed the COVID-19 assessment (right y-axis, gray bars). The shaded region shows the dates (April 27th, 2020 and May 15th, 2020) during which participants completed the COVID-19 assessment. All COVID-19 statistics were obtained daily from the Centers for Disease Control and Prevention COVID-19 Data Tracker (https://covid.cdc.gov/covid-data-tracker/#trends_dailytrends).

Adolescents and young adults participating in two ongoing longitudinal studies were assessed between December 2014 and July 2019, and then again during the COVID-19 outbreak in New York between March 27^th^, one week after COVID-19 was declared a pandemic by the World Health Organization (2020), and May 15^th^ 2020. Figure 1 displays the timing of study participation in reference to incidence of new COVID-19 cases in New York state. As the figure demonstrates, data collection spans the peak period of COVID-19 infection in New York, with particularly heavy sampling during the initial escalation in cases. We assessed change in symptoms from before to during the pandemic, and explored the impact of various pandemic-related experiences on symptom change.

## Methods

### Participants

The sample included 451 adolescents and young adults who participated in one of two longitudinal investigations at Stony Brook University.

#### Impact of puberty on affect and neural development across adolescence (iPANDA)

The iPANDA project is a longitudinal investigation of developmental trajectories of neural reward sensitivity and psychopathology in a community sample of 317 girls from Long Island, New York (Burani et al., 2019). Participants were initially recruited using a commercial mailing list of families with an 8- to 14-year-old daughter living in a 20-mile radius of Stony Brook University, posted flyers, online postings, and word of mouth. Families were eligible to participate if they had a daughter aged between 8 and 14 with no known medical or developmental disability, a biological parent willing to participate, and the ability to read and write English. Following the first (i.e. baseline) wave of data collection (ages 8–14), participants were reassessed at two-year intervals (i.e. ages 10–16 for the second wave and 12–18 for the third wave). At time of the COVID-19 pandemic, the iPANDA project was in the middle of the third wave (ages 12–18) and not all participants had completed this assessment. Therefore, the pre-COVID-19 assessment was identified as the second wave of data collection (ages 10–16; *n* = 263, 83% of the baseline sample).

#### Stony Brook Temperament Study (SBTS)

The SBTS is a longitudinal study designed to explore early precursors to depression and anxiety disorders in a community sample of youth from Long Island, NY (Klein & Finsaas, 2017). Participants (*N* = 559) were recruited using a commercial mailing list of families with a 3-year-old child living in a 20-mile radius of Stony Brook University. Eligibility criteria included having a 3-year-old child with no significant medical disorder or developmental disability and a biological parent with the ability to read and write English. Following the age 3 assessment, families were invited to participate in follow-up assessments when their child was approximately age 6, 9, 12, 15, and 18. To increase the diversity of the sample, 50 children from underrepresented minority groups were added to the sample in the age 6 wave. At the time of the COVID-19 pandemic, the SBTS had just begun its sixth wave (age 18) of data collection. Therefore, the pre-COVID-19 assessment was identified as the fifth wave of data collection (age 15; *n* = 450, 73.9% baseline sample).

### Measures

#### Children's depression inventory (CDI)

The CDI is a self-report questionnaire designed to assess depression symptoms occurring over the past 2 weeks in children and adolescents aged 7–17 years (Kovacs, 1992). To maintain continuity across participants and with prior assessment waves, the CDI was administered to all participants regardless of their current age. The CDI contains 27 items rated on a 0–2 scale. Items are summed to create a composite measure of current depression symptoms, with higher scores reflecting greater severity. The CDI has been shown to be a reliable and valid measure of depression symptoms in youth (Dougherty, Klein, & Olino, 2018).

#### Screen for child anxiety-related disorders (SCARED)

The SCARED is self-report measure of anxiety disorder symptoms over the past month in children and adolescents aged 8–18 years (Birmaher et al., 1997). It contains 41 items that are rated on a 3-point scale: *almost never* (*0*), *sometimes* (*1*), and *often* (*2*). There are 5 subscales that measure different anxiety symptoms that parallel anxiety disorders classified in the DSM, including panic/somatic symptoms, generalized anxiety, separation anxiety, social anxiety, and school phobia. The SCARED has demonstrated adequate psychometric properties in both clinical and non-clinical samples (Rappaport, Pagliaccio, Pine, Klein, & Jarcho, 2017). Similar to the CDI, the SCARED was administered to all participants regardless of current age to maintain continuity across participants and with prior assessment waves. The present study focused on the panic/somatic symptoms, generalized anxiety, and social anxiety subscales.

#### Pandemic experiences survey

We created a survey for the COVID-19 assessment to capture experiences related to the pandemic, drawing on measures developed to assess the impact of prior natural and man-made disasters on everyday life (e.g. for Hurricane Ike and Hurricane Katrina; Goldmann & Galea, 2014; Norris, Sherrieb, & Galea, 2010) and personal accounts reported in local and national news. Due to the rapidly evolving impact of the pandemic on daily life, some of the items developed for the survey became less relevant between the time of survey development and administration (e.g. avoiding public places was endorsed by almost all participants). The remaining 26 items were combined to create 5 composites capturing different domains of experience related to the pandemic: life changes, infection concerns, school concerns, home confinement concerns, and basic needs concerns. All 26 items are listed in Table A1 in the Appendix.

The life changes composite included a checklist of 14 changes in life circumstances related to the pandemic (e.g. job moved online, forced to change where you live). Scores ranged from 0 to 8 (*M* = 3.48, *s.d.* = 1.61). The infection concerns composite included two items probing concern about and perceived likelihood of becoming infected with COVID-19, rated on a Likert-scale from 0 (*not at all*) to 4 (*extremely*). Scores ranged from 0 to 8 (*M* = 3.03, *s.d.* = 1.52; Cronbach's alpha = 0.62). The school concerns composite included a checklist of four school-related concerns (e.g. online classes being lower quality). Scores ranged from 0 to 4 (*M* = 1.25, *s.d.* = 1.15; Cronbach's alpha = 0.76). The home confinement concerns composite included a checklist of three concerns related to being largely restricted to home (e.g. experiencing cabin fever). Scores ranged from 0 to 3 (*M* = 1.32, *s.d.* = 0.95; Cronbach's alpha = 0.73). Finally, the basic needs concerns subscale included a checklist of three concerns related to having one's basic needs met (e.g. not having enough food or supplies). Scores ranged from 0 to 3 (*M* = 0.57, *s.d.* = 0.79; Cronbach's alpha = 0.71).

### Procedure

Participants completed the pre-COVID-19 CDI and SCARED assessment between December 2014 and August 2017 for the iPANDA project and July 2016 and July 2019 for the SBTS. All participants completed the COVID-19 CDI, SCARED, and pandemic experiences assessment between March 27th, 2020 and May 15th, 2020. All assessments were completed using survey software on a computer. Pre-COVID-19 assessments were completed either on a lab computer during the lab visit or at-home on the participant's personal device, while all COVID-19 assessments were completed at-home on the participant's personal device.

### Data analysis

Adolescence is a critical period for the emergence and exacerbation of depression and anxiety symptoms (Gruber et al., 2020), so it is possible that increases in symptoms from the pre-COVID to the COVID-19 assessments could be part of a developmentally normative process. Therefore, age at the pre-COVID-19 and COVID-19 assessments, which were moderately correlated, Pearson's *r*(451) = 0.47, *p* < 0.001, were included as covariates in all analyses. Controlling for age at both assessments allowed us to account for not only age effects but also differences in the interval between assessments. Change in CDI and SCARED symptoms was examined via repeated measures analysis of covariance (ANCOVA) with time (pre-COVID-19 *v.* COVID-19) as a within-subject factor and age at the pre-COVID-19 and COVID-19 assessments as covariates. Repeated measures ANCOVA allowed us to test changes in symptoms as a function of time, while controlling for continuous covariates (age). Gender difference in symptom change was examined via mixed-design ANCOVA with time (pre-COVID-19 *v.* COVID-19) as a within-subject factor and gender [females (1) *v.* males (0)] as a between-subjects factor, and age at the pre-COVID-19 and COVID-19 assessments as covariates. Cohen's *d*, an effect size index of the magnitude of difference between means, is reported for symptom changes pre-COVID-19 to COVID-19 (*d* = 0.2, 0.5 and 0.8 are considered small, medium and large, respectively).

In addition to the reporting of mean symptom scores, estimated rates of psychiatric disorders were examined using established clinical cutoffs. For both the CDI and SCARED, scores were first converted to *T*-scores based on normative samples for each measure (Kovacs, 1992; Rappaport et al., 2017). Next, the percentage of participants with clinically elevated symptoms was determined based on cutoffs corresponding to a *T*-score ⩾65, which included a CDI total score ⩾19 in females and ⩾24 in males and SCARED scores of panic/somatic symptoms ⩾9, generalized anxiety ⩾9, and social anxiety ⩾8.

Associations between the pandemic experiences composites and change in CDI and SCARED symptoms was examined with a three-step analytical approach. First, CDI and SCARED symptom residuals were created by regressing COVID-19 symptoms on pre-COVID-19 symptoms. Residuals were used instead of raw change scores, as the latter is liable to problems with reliability and regression to the mean. Next, partial correlations were computed between the pandemic experiences composites and COVID-19 symptom residuals, adjusting for age at the pre-COVID-19 and COVID-19 assessments. Finally, multivariable linear regression was conducted to examine *unique* associations between the pandemic experiences composites and each CDI and SCARED symptom residual. Specifically, age at the pre-COVID-19 and COVID-19 assessments, gender [females (1) *v.* males (0)], all other CDI and SCARED symptom residuals, and all pandemic experiences composites were included as independent variables, and the CDI or SCARED symptom residual of interest served as the dependent variable. Separate analyses were conducted for each CDI and SCARED residual as the dependent measure. All analyses were conducted in IBM SPSS Statistics 26.0.

## Results

### Demographics

Among the 713 participants who completed the pre-COVID assessment, 505 (70.8%) participated in the COVID-19 assessment, and 54 were missing symptom or pandemic experience data. Little's MCAR test suggested that listwise deletion of participants with incomplete COVID-19 assessment data was appropriate, χ^2^(101) = 102.03, *p* = 0.43 (Little, 1988). The final sample comprised 45 112 to 22-year-old participants (*M* = 17.49, *s.d.* = 1.42), including 295 (65.4%) females and 374 (82.9%) individuals who identified as White/non-Hispanic. Participant educational background included 305 (67.6%) who were currently in high school and 141 (31.3%) who were currently in college. Parental educational background included 289 (64.1%) participants with a parent who was a college graduate.

Attrition analyses indicated that participants included in the final analyses (*n* = 451), relative to those who did not complete the COVID-19 assessment or were excluded from analyses (*n* = 262), were more likely to be female, White/non-Hispanic, 74.8% *v.* 64.4%, χ^2^(1) = 6.81, *p* = 0.01, and have a parent who was college educated, 53.8% *v.* 49.2%, χ^2^(1) = 7.63, *p* = 0.01. Groups did not differ in age at the pre-COVID-19 assessment, *t*(711) = −0.48, *p* = 0.63.

### Psychiatric symptoms

Table 1 displays means (and standard deviations) for CDI and SCARED symptoms before and during the COVID-19 pandemic. Across all participants and independent of age, psychiatric symptoms increased during the pandemic, including depression, panic/somatic symptoms, generalized anxiety, and social anxiety. Effect sizes ranged from small to moderate in magnitude. Gender analyses indicated a significant Time × Gender interaction for depression, *F*(1,447) = 6.22, *p* = 0.013 and panic/somatic symptoms, *F*(1,447) = 4.80, *p* = 0.029, but not generalized or social anxiety symptoms. *Post hoc* analyses reveal that depression and panic/somatic symptoms increased for females only.

**Table 1.** Depression and anxiety symptoms before and during the COVID-19 pandemic

	Pre-COVID-19 *M* (*s.d.*)	COVID-19 *M* (*s.d.*)	*F*	*p*	Cohen's *d*
CDI	5.93 (5.70)	9.61 (7.74)	10.11	0.002	0.29
Females	6.49 (9.22)	10.76 (8.23)	10.13	0.002	0.34
Males	4.68 (4.09)	7.05 (5.78)	0.94	0.34	<0.01
SCARED					
Panic/Somatic symptoms	4.77 (4.29)	4.96 (5.03)	13.45	<0.001	0.33
Females	5.27 (4.68)	5.91 (5.41)	12.21	0.001	0.38
Males	3.64 (3.01)	2.86 (3.15)	0.01	0.99	<0.01
Generalized anxiety	5.62 (4.38)	7.62 (4.92)	20.58	<0.001	0.42
Social anxiety	4.83 (3.57)	5.43 (4.00)	7.57	0.006	0.24

Note. Age at the pre-COVID-19 and COVID-19 assessments were included as covariates in all analyses. Gender-specific tests are only reported where there was a significant Time × Gender interaction. CDI = Children's Depression Inventory; *M* = mean; SCARED = Screen for Child Anxiety Related Disorders; *s.d.* = standard deviation.

### Clinical range symptoms

**Fig. 2.**
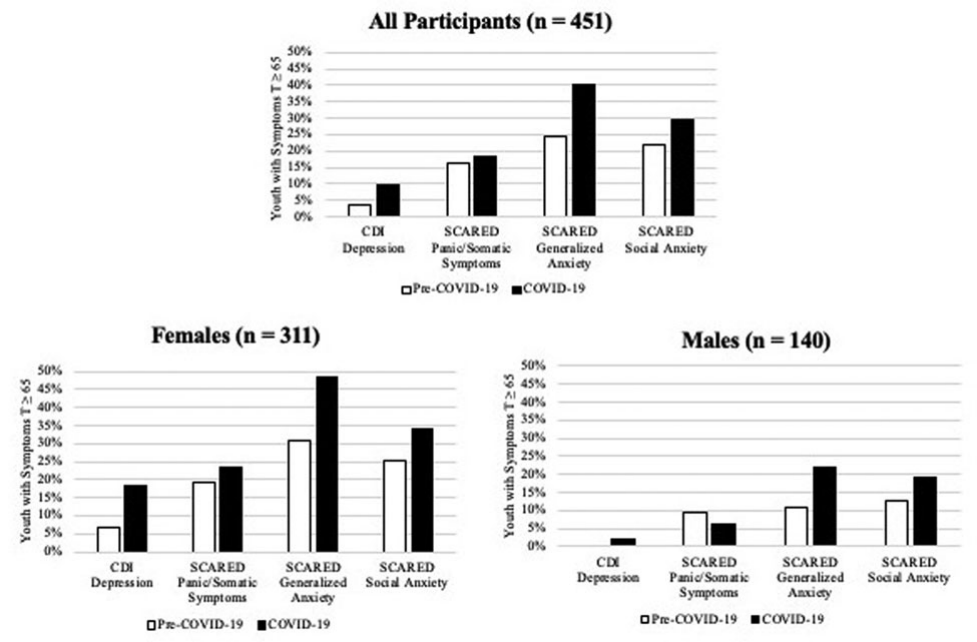
Percentage of participants with CDI or SCARED symptoms in the clinical range (*T*⩾65). CDI = Children's Depression Inventory; SCARED = Screen for Child Anxiety Related Disorders.

Figure 2 displays the percentage of participants with CDI or SCARED symptoms in the clinical range. The percentage of participants with clinically elevated depression, panic/somatic symptoms, generalized anxiety and social anxiety symptoms during COVID-19 was 10.4%, 18.2%, 40.4%, and 29.5%, respectively. In female participants, there was a nearly three-fold increase in rates of clinically elevated depression from pre-COVID-19 to COVID-19 and nearly half (49%) experienced clinically elevated generalized anxiety during COVID-19.

### Pandemic experiences

Table 2 displays partial correlations between the five pandemic experiences composites and CDI and SCARED symptom residuals. Greater COVID-19 life changes and infection, school, home confinement, and basic needs concerns were associated with increased depression, panic/somatic symptoms, and generalized anxiety symptoms. Greater COVID-19 infection and school concerns were associated with increased social anxiety symptoms.

**Table 2.** Partial correlations between COVID-19 experiences and change in depression and anxiety symptoms

	Pandemic experiences				
Symptoms	Life changes	Infection concerns	School concerns	Home confinement concerns	Basic needs concerns
CDI	0.18***	0.17***	0.32***	0.18***	0.19***
SCARED					
Panic/Somatic symptoms	0.13**	0.11*	0.16***	0.09*	0.16***
Generalized anxiety	0.17***	0.20***	0.18***	0.20***	0.22***
Social anxiety	0.05	0.10*	0.11*	−0.01	0.08

Note. CDI and SCARED residuals were computed by regressing COVID-19 symptoms on pre-COVID-19 symptoms. Partial correlations controlled for age at the pre-COVID-19 and COVID-19 assessments. CDI = Children's Depression Inventory; SCARED = Screen for Child Anxiety Related Disorders.

* *p* < 0.05, ** *p* < 0.01, *** *p* < 0.001.

Table 3 displays results for the multivariable linear regression analyses. Greater COVID-19 school concerns were uniquely associated with increased depression symptoms. Greater COVID-19 home confinement concerns were uniquely associated with increased generalized anxiety symptoms. Finally, home confinement concerns were uniquely associated with decreased social anxiety symptoms. None of the composites were uniquely associated with panic/somatic symptoms. Gender did not moderate any relationship between pandemic experiences and change in CDI or SCARED symptoms.

**Table 3.** Coefficients for independent relationship between COVID-19 experiences and change in depression and anxiety symptoms

	Pandemic experiences				
Symptoms	Life changes	Infection concerns	School concerns	Home confinement concerns	Basic needs concerns
	*B*(s.e.), *β*	*B*(s.e.), *β*	*B*(s.e.), *β*	*B*(s.e.), *β*	*B*(s.e.), *β*
CDI	0.13(0.16), 0.03	0.04(0.17), 0.01	1.08(0.22), 0.19***	0.26(0.25), 0.04	0.02(0.32), 0.00
SCARED					
Panic/Somatic symptoms	0.06(0.12), 0.02	−0.10(0.12), −0.03	0.03(0.16), 0.00	−0.14(0.18), −0.03	0.11(0.23), 0.02
Generalized anxiety	0.04(0.09), 0.02	0.18(0.09), 0.07	−0.17(0.13), −0.05	0.46(0.14), 0.11**	0.31(0.18), 0.06
Social anxiety	−0.07(0.09), −0.04	0.02(0.09), 0.01	0.08(0.12), 0.03	−0.36(0.14), −0.11*	−0.06(0.18), −0.01

Note. CDI and SCARED residuals were computed by regressing COVID-19 symptoms on pre-COVID-19 symptoms. Multivariable linear regressions were conducted separately for each CDI and SCARED symptom. Age at the pre-COVID-19 and COVID-19 assessments, gender (females (1) v. males (0)), all other CDI and SCARED residuals, and all pandemic experiences composites were included as independent variables in each multivariable linear regression. The CDI and SCARED residual of interest was included as the dependent variable. Each row displays the B = unstandardized coefficients (s.e. = standard error), and *β* = standardized beta coefficients for pandemic experiences composites as independent variables for a single multivariable linear regression. CDI = Children's Depression Inventory; SCARED = Screen for Child Anxiety Related Disorders.

* *p* < 0.05, ** *p* < 0.01, *** *p* < 0.00.

We conducted sensitivity analyses controlling for study and all demographic variables. None of the results changed substantively when accounting for these variables.

## Discussion

Prior studies using nationally representative samples have suggested that incidence of depression and anxiety increased following the COVID-19 pandemic in the U.S., which has been particularly impacted (Ettman et al., 2020; Twenge & Joiner, 2020). A handful of studies have explored within-person increases in symptoms of depression and anxiety in youth (Elmer et al., 2020; Li et al., 2020; Magson et al., 2020; Saraswathi et al., 2020), however, just one of these studies was conducted in the U.S. (Lee et al., 2020). Our findings provide insight into the specific pandemic experiences that contribute to worsening mental health in youth, whereas prior research has primarily been restricted to non-specific measures of increased stress or demographic moderators of risk, often at times and in regions that were not severely affected by COVID-19.

Findings of the current study suggest that the COVID-19 pandemic contributed to increased symptoms of generalized and social anxiety in youth living in Long Island, New York. Moreover, females also experienced increased depression and panic/somatic symptoms. Depression and anxiety typically increase from late childhood through early adulthood (Gruber et al., 2020). However, our sample spanned a broad age range (12–22), and we controlled for age at both assessments, so it is unlikely that these results can be attributed to normative developmental processes. Effect sizes for symptom increases were small to medium, indicating that average levels of symptom change were modest, but high rates of clinically elevated symptoms suggest that many youth are struggling during the COVID-19 pandemic.

Consistent with research on prior outbreaks (Wenham et al., 2020), females appear to be particularly vulnerable to experiencing increased mental health problems during COVID-19. In our sample, only females saw increases in depression and all three types of anxiety symptoms, and nearly 60% of females met the clinical cut-off for at least one disorder during COVID-19. These sex differences could be due to greater exposure to stressors during the pandemic and/or heightened response to stress in females. A large literature suggests that females are more likely to develop internalizing symptoms following exposure to stress and trauma, even accounting for the specific event (Tolin & Foa, 2008).

Across domains, pandemic experiences were associated with increases in psychiatric symptoms. Specifically, greater concern about contracting COVID-19 and school-related problems were associated with increases in both depression and all three types of anxiety symptoms, while experiencing more life changes and greater concern about being confined at home and meeting basic needs were associated with increased depression, panic/somatic symptoms and generalized anxiety. Multivariable analyses suggest that school concerns, including concerns about passing classes, juggling schoolwork with other responsibilities and online classes being poor quality, were uniquely associated with increases in depression symptoms. This is consistent with a study on adolescents in Australia during COVID-19 (Magson et al., 2020) and suggests that the transition to online learning may have been a particularly significant stressor for youth. Concerns related to being confined at home, including experiencing cabin fever and a limited social life, were uniquely associated with increases in generalized anxiety, which suggests that shelter-in-place and social distancing measures more specifically contribute to increased diffuse worries. Finally, after adjusting for anxiety and other depressive symptoms, home confinement was uniquely associated with *decreased* social anxiety symptoms, indicating that, for some youth, the pandemic has provided a respite from social pressures. Further work is needed to determine how this will impact social development and anxiety after the pandemic.

This study possesses many strengths, including a longitudinal design capturing within-person changes in symptoms, assessment of a range of pandemic experiences, and a sample residing in one of the largest epicenters of the pandemic at the time of assessment. However, there were several limitations. The attrition rate raises concerns about generalizability of these findings. Participant recruitment was non-random, although the demographic make-up of the baseline sample is similar to census data for Suffolk County, NY (U.S. Census Bureau, 2019). Additionally, sensitivity analyses suggest that findings do not change substantively when accounting for sample demographics related to attrition. Due to the importance of conducting assessments in a short period of time, we had to use symptom inventories instead of diagnostic interviews. Timing of the pre-COVID-19 assessment and the interval between assessments varied substantially, although we controlled for age at both assessments to account for these differences. Follow-up is needed to determine the persistence of these effects over the course of the pandemic. Finally, without a demographic and time-matched control group not impacted by the pandemic, we cannot establish whether symptom increases were causally related to the COVID-19 pandemic.

## Appendix

**Table A1.** Pandemic experiences composites

**Life Changes** In the last month, have you been impacted by the novel coronavirus (COVID-19) pandemic in any of the following ways? (check all that apply) 1. Education moved to online instruction 2. Job/occupation/work moved to at home/remote/online 3. Reduced hours or laid off from work 4. Working extra hours at work 5. Forced to change where you live 6. Relatives or others have moved into where you live 7. Mandated quarantine (e.g. due to traveling or contact with someone with the virus) Have you experienced or are you expecting a reduction in personal or family income? 8. Yes, some or a substantial reduction Has the coronavirus affected the level of conflict or problems with your family? 9. There is somewhat more, or much more, conflict/problems Has the coronavirus affected how close you are to your friends? 10. We are somewhat less, or much less, close Has the coronavirus affected the level of conflict or problems with your friends? 11. There is somewhat more, or much more, conflict/problems In the last month, do you know anyone who has tested positive for coronavirus (COVID-19)? 12. Yes (Family member, romantic partner, friend, roommate or co-worker) In the last month, have you or has anyone close to you been hospitalized due to coronavirus (COVID-19)? 13. Yes (Family member, romantic partner, friend, roommate or co-worker In the last month, has anyone close to you died due to coronavirus (COVID-19)? 14. Yes (Family member, romantic partner, friend, roommate or co-worker)
**Infection Concerns** 1. How concerned are you about becoming infected with the coronavirus (COVID-19)? (multiple choice) a. Not at all b. A little bit c. Moderately d. Quite a bit e. Extremely 2. How likely do you think it is that you could become infected with the coronavirus (COVID-19)? (multiple choice) a. Not at all b. A little bit c. Moderately d. Quite a bit e. Extremely
**School Concerns** How is your schoolwork going? 1. Poorly or very poorly Which of the following are your biggest concerns (besides you getting or having the coronavirus) in the last month: (check all that apply) 1. Juggling school/work responsibilities with care-giving duties 2. Not being able to finish classes with passing grades 3. Online classes will be lower quality giving me a weak background as I continue into upper division courses or graduate
**Home Confinement Concerns** Which of the following are your biggest concerns (besides you getting or having the coronavirus) in the last month: (check all that apply) 1. Cabin fever 2. Limited or no social life 3. Being stuck living in a small space
**Basic Needs Concerns** Which of the following are your biggest concerns (besides you getting or having the coronavirus) in the last month: (check all that apply) 1. Not having enough food to eat 2. Reduced family income 3. Not having enough paper products or cleaning products
